# A novel compound heterozygous mutation of the *SMARCAL1* gene leading to mild Schimke immune-osseous dysplasia: a case report

**DOI:** 10.1186/s12887-017-0968-8

**Published:** 2017-12-28

**Authors:** Shuaimei Liu, Mingchao Zhang, Mengxia Ni, Peiran Zhu, Xinyi Xia

**Affiliations:** 1 Department of Reproduction and Genetics, Institute of Laboratory Medicine, Jinling Hospital, Nanjing University School of Medicine, Nanjing, 210002 People’s Republic of China; 2 National Clinical Research Center of Kidney Diseases, Jinling Hospital, Nanjing University School of Medicine, Nanjing, 210016 People’s Republic of China

**Keywords:** Schimke immune-osseous dysplasia, *SMARCAL1*, Next generation sequencing, Mutation analysis

## Abstract

**Background:**

Schimke immune-osseous dysplasia (SIOD, OMIM 242900) is characterized by spondyloepiphyseal dysplasia, T-cell deficiency, renal dysfunction and special facial features. *SMARCAL1* gene mutations are determined in approximately 50% of patients diagnosed with SIOD.

**Case presentation:**

The case presented here is that of a 6-year-old boy who was born at 33 weeks to healthy, non-consanguineous Chinese parents. He presented with short stature (95 cm; <3rd percentile) and proteinuria. Initially suspected of having IgM nephropathy, the patient was finally diagnosed with mild Schimke immune-osseous dysplasia. One novel mutation (p.R817H) and one well-known mutation (p.R645C) was identified in the *SMARCAL1* gene.

**Conclusion:**

This report describes a clinical and genetic diagnostic model of mild SIOD. It also highlights the importance of molecular testing or clinical diagnosis and the guidance it provides in disease prognosis.

## Background

Schimke immune-osseous dysplasia (SIOD, MIM 242900) is characterized by spondyloepiphyseal dysplasia (SED), T-cell deficiency, renal dysfunction and special facial features [1–3]. SIOD is a rare, multi-system, autosomal recessive disease with an incidence of 1:1 × 10^6^~3 × 10^6^. SIOD manifests in approximately 50% of patients due to mutations in the *SMARCAL1* gene. Maintaining DNA stability, DNA replication, and recombination or DNA repair, *SMARCAL1* (SWI/SNF-related, matrix associated, actin-dependent regulator of chromatin, subfamily a-like 1) is a member of the SNF2 family [4, 5]. SIOD disease severity is determined by different types of *SMARCAL1* mutations. *SMARCAL1* nonsense, frame shift and splicing mutations can lead to severe clinical manifestations. Contrarily, most missense mutations cause mild symptoms.

SIOD was first reported in 1971 [6], and its phenotype varies from mild to severe [7, 8]. Nonsense, frame shift and splicing mutations in the *SMARCAL1* gene destroy the normal structure of SNF2 proteins, consequently producing truncated protein products. Several homozygous/heterozygous missense mutations lead to a severe phenotype [2]. Contrary to this, a large number of bi-allelic missense mutations are associated with mild clinical symptoms. No significant differences have been described between the two types of clinical manifestations. Patients with mild SIOD can survive into adulthood with reasonable treatment [9]. Severe phenotypes result in death in juvenile patients, ultimately after the development of end stage renal disease.

Here, the case of a 6-year-old boy with mild SIOD is presented. Next-generation sequencing technology was applied to samples collected from this patient in order to investigate the *SMARCAL1* gene and potentially identify pathogenic mutations.

## Case presentation

The patient, a 6-year-old boy, is the first child born to healthy, non-consanguineous, Chinese parents. Initially admitted to the People’s Hospital of Human Province due to short stature (95 cm; < 3rd percentile), he was later referred to Nanjing Jinling Hospital at 5.7 years of age as the patient had experienced proteinuria over the course of 3 months. Born prematurely at 33 weeks, his birth weight was 1.96 kg (< 3^rd^percentile).

**Fig. 1:**
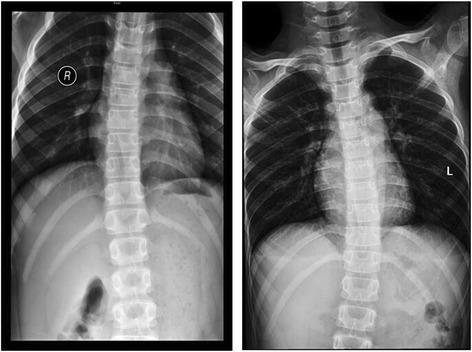
The spine radiograph showing the litter patient has scoliosis

Laboratory investigations revealed routine urine protein concentration of 3+, a white blood cell count of 10.1/L (3.5–9.5 × 10^9^/L), and a lymphocyte percentage of 10.52% (20%–50%). Serum biochemical measurements showed the following concentrations: total protein59g/L (65.0–85.0 g/L), albumin 31.8 g/L (40.0–55.0 g/L), urea 2.6 mmol/L (2.9–8.2 mmol/L), creatinine 23 μmol/L (53–123 μmol/L), total cholesterol 7.63 mmol/L (<5.18 mmol/L), and triglycerides 2.61 mmol/L (<1.70 mmol/L). T and B lymphocyte subset analysis revealed the following: B cells constituting 36.7% (6.4%–22.6%), NK cells comprising 11.3% (5.6%–30.9%), a CD3+ T-lymphocyterate of 35.6% (61.1%–77%), a CD3+ CD4+ T-lymphocyte frequency of 10.2% (15.8%–41.6%), and a CD3+ CD8+ T-lymphocyte presence of22.5% (18.1%–29.6%) within the sample. Showing a congenital immune deficiency, decreased blood IgG values were observed. Renal biopsy analysis revealed the presence of 37 glomeruli, while immunohistochemical studies indicated positive capillary wall IgA, IgM, IgG values and mild, partial glomerular segmental mesangial matrix hyperplasia. Pathologically, this led to the diagnosis of IgM nephropathy. After having been prescribed immunosuppressive treatment of 10 mg prednisone TID, urine protein concentrations dropped to 2+. Non-negative urine protein effects were observed with the administration of methylprednisolone and cyclophosphamide pulse therapy (specific dose is unknown). Proteinuria was significantly positive, and showed the presence of glomerulus albuminuria. In order to further establish a diagnosis and treatment regimen, the patient was transferred to the nephritic department at the Nanjing Jinling Hospital. Physical examination found that the patient exhibited normal facial expression, had normal skull structure and thyroid function, was of normal intelligence. However, it’s worth mentioning that spine of the litter patient has scoliosis (Fig. 1). A deficiency of growth hormones was not identified. However, the patient did experience puffy eyelids and edema of the lower extremities. Retinitis pigmentosa was not detected. Both parents were found to be phenotypically normal. Therefore, under the consent of the patient and his family, next generation sequencing was used to perform genetic testing. On the basis of clinical and laboratory findings, the diagnosis of SIOD is doubtful.

## Discussion and conclusions

SIOD is an autosomal-recessive, multisystem disorder with a low incidence. So far, only one pathogenic gene, *SMARCAL1*, has been associated with SIOD. The *SMARCAL1* gene is located on chromosome 2q34-q36, and contains 18 exons. Exon1 and 2 do not participate in protein coding, while the remaining exons encode the 954aa protein. Due to the conveniently short sequence that is generated, many researchers choose different methods to detect potential *SMARCAL1* gene mutations. Zivicnjak [10] used direct sequencing in search of novel compound mutations of *SMARCAL1* in two female siblings, while Simon [11] reported novel *SMARCAL1*bi-allelic mutations by employing whole-exome sequencing methods. Carroll [12] discovered a novel splice site mutation in *SMARCAL1* through next generation sequencing (NGS). In this study, NGS was used to screen for, and Sanger sequencing to verify, the presence of SIOD mutations. Several mutations associated with the manifestation of SIOD have been found. However, current methods failed to detect variants causative of SIOD in approximately 50% of diagnosed patients. It is suspected that this may be associated with the following factors: 1) deep intronic region mutations, 2) some pathogenic genes have not been discovered and/or described, 3) environmental factors can modify the gene expression [13], and 4) endophenotypes may potentially exist [3].

SIOD shows phenotypic heterogeneity [11], and disease severity varies from mild to severe. SIOD patients with a severe phenotype typically die before the age of five and are characterized by osseous dysplasia, hypermicrosoma, special facial dysmorphism, and T cell deficiency caused by repeated infection and chromosomal fragility [14]. There are truncating *SMARCAL1*mutations (nonsense, frame shift and splicing mutations) that result in a severe disease phenotype. On the other hand, when compared to severe SIOD patients, mild SIOD patients manifest symptoms that are slower to progress in severity. Some may present without infections, and are sometimes clinically asymptomatic, with no proteinuria detected in the early-childhood onset cases. Mild SIOD patients generally survive up to the age of 15 years, while some patients may survive beyond 36 years of age [15]. This case describes that of a 6-year-old boy with clinically mild manifestations. After a 1 year follow-up examination, the clinical situation of the patient had improved. It is worth mentioning that the patient’s proteinuria had disappeared. Taking advantage of next-generation sequencing, two *SMARCAL1*missense mutations were discovered in this patient. Boerkoel [1] reported the genotypes present in three families with the milder form of SIOD. One family had compound heterozygous mutations (I548N, R645C), while the R586W, and K647 T mutations were respectively identified in homozygotic states in the remaining two families. The mild clinical phenotype found in this patient corresponds exactly with that described by Boerkoel [1]. All of the affected individuals were short in stature, and had renal disease and lymphocytopenia, while lacking recurrent infections. It is noteworthy that affected individuals described in previous studies were all more than 15 years of age after undergoing renal transplantation. The patient presented in this study had a mild clinical phenotype but had not yet undergone renal transplantation. This milder phenotype caused by missense mutations may be due to residual *SMARCAL1* function [1]. However, Yue [16] and Jimena [17] have reported compound heterozygous affected individuals presenting with a severe phenotype due to missense mutations. These differences may be attributed to environmental or genetic influences. The presence of missense mutations is therefore unlikely to accurately predict disease phenotype.

**Fig. 2:**
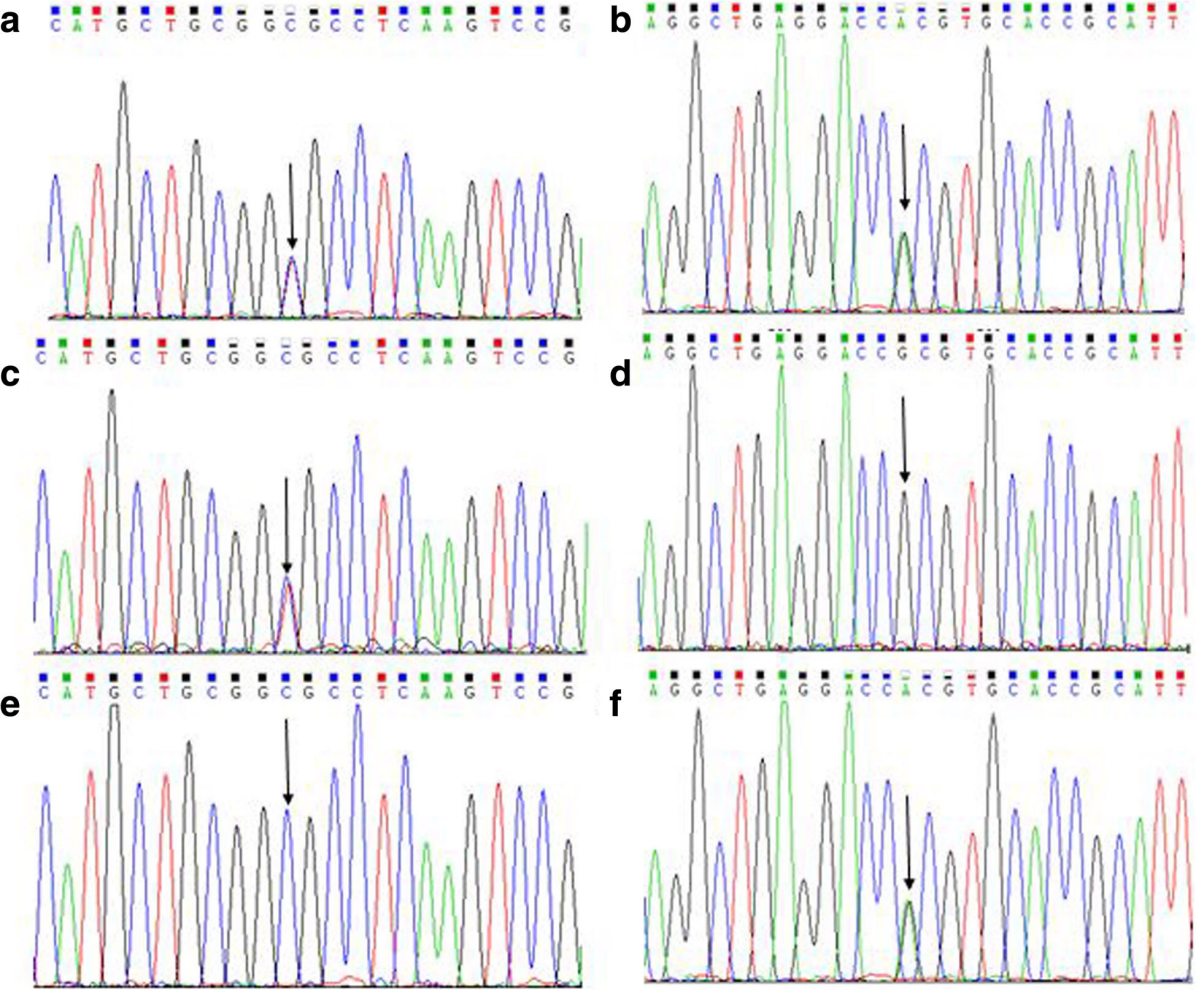
Genetic analysis of the family. Mutations analysis: the patient carries two mutations (**a** and **b**) of *SMARCAL1* gene. The mother carries the c.1933C > T mutation (**c** and **d**) and the father carries the c.2450G > A mutation (**e** and **f**). Arrows indicate the position of the mutations

**Fig. 3:**
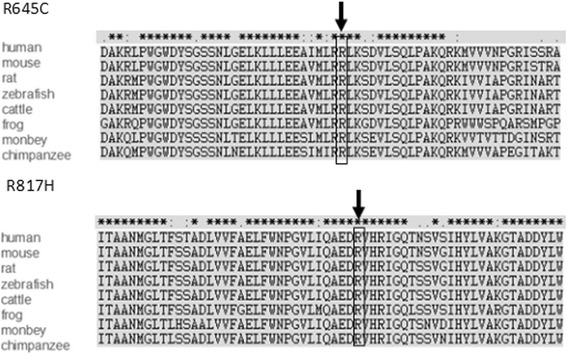
Multi-sequence alignments of *SMARCAL1* protein shows invariance of R645C and R817H from human to chimpanzee. In silico analysis of the likely pathogenicity of the two mutations shows variant scores (SIFT = 0.00, PolyPhen-2 = 1.00) characteristic of a highly likely pathogenic mutations. The red box indicated the positions of SMARCAL1 mutatnt proteins

The patient described in this study harbored a paternally-derived missense mutation (c.2450G > A) in exon 16 of *SMARCAL1* leading to an arginine-to-histidine substitution (Fig. 2). Resulting in an arginine-to-cysteine substitution, the patient also presented with a well-known maternally inherited missense mutation (c.1933C > T) [9] in exon 12 of the*SMARCAL1* gene. Several explanations exist to describe the arginine-to-histidine amino acid change at position 817. Regardless, the two mutated sites are highly conserved in the house mouse, Norway rat, zebra fish, cattle, frog, monkey, and chimpanzee animal models. Described for the first time in our report, the missense mutation (p.R817H) is located in the DNA/RNA helicase C-terminal domain of the protein. It is forecasted to be detrimental to the patient with a score of 0.0 by employing the Sorting Intolerant from Tolerant (SIFT, http://sift.jcvi.org/) technique. Similarly, the potential effect of substitution has a detrimental score of 1.000 as calculated by PolyPhen-2 (http://genetics.bwh.harvard.edu/pph2/) (Fig. 3).

**Fig. 4:**
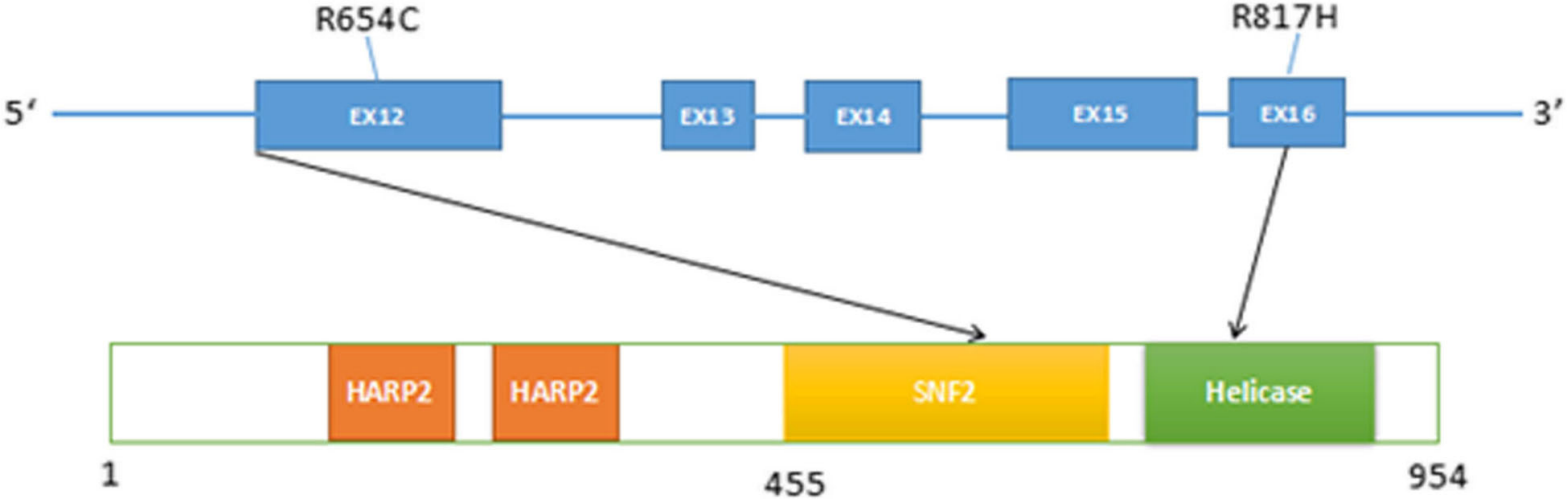
Schematic diagram of *SMARCAL1* gene. Functional structure domains of *SMARCAL1*gene from exon 12 to exon 16 which contains mutant sites (R654C and R817H) of our report, respectively. Orange represents HARP2 domains, yellow is symbolic of SNF2 N-terminal domain, green stands for DNA/RNA helicase C-terminal domain

*SMARCAL1* is a replication stress response and single strand DNA binding protein [9]. As an ATP-dependent annealing helicase, this protein contains two DNA/RNA HARP2 helicases at the C-terminal, and has a SNF2 N-terminal domain. *SMARCAL1* catalyzes the rewinding of the stably unwound DNA. SNF2-related proteins are distinguished by the presence of SWI/SNF helicase motifs (I, Ia, II, III, IV, V and VI). DNA/RNA helicases partake in nucleotide triphosphate hydrolysis and in the coupling of DNA binding [18–20]. Correlated to*SMARCAL1*gene mutations, multiple mechanisms could bring about the loss of functional proteins in SIOD patients [21]. Missense mutations in the *SMARCAL1* SNF2 domain decreases DNA-dependent ATPase activity [21]. To date, the common missense mutations R586W, R645C and R820H have all been detected in the conserved arginine residues of the SMARCAL1 protein. Mutations R586W andR820H belong to a region associated with DNA binding and ATPase activity. Since the novel R817H mutation detected in this study is located adjacent to the R820H mutation found within the DNA/RNA helicase domain, the R817H variant may similarly affect ATPase function through altering the *SMARCAL1* structure or protein interaction capacity. The known missense mutation R645C is located in the SNF2 domain and is associated with putative nuclear localization. It is predicted to interfere with the mobility of the hinge region and prevent competent clamping of *SMARCAL1* on the DNA [22].This is similar to the effects observed with the R644W, K647Q, and K647 T mutations. *SMARCAL1* mutations result in cell proliferation defects and a promotion of apoptosis. SMARCAL1-deficient zebrafish were associated with growth retardation and defects in hematopoiesis [23]. Growth failure caused by skeletal dysplasia in SIOD patients is not as a result of renal disease. The functional loss of SMARCAL1 in SIOD patients contribute to multiple phenotypes resulting from the instability of DNA replication throughout the genome [24]. In an vitro study, Marie [25] reported that a deficiency of SMARCAL1 altered the chromatin structure, thereby affecting gene expression. Recently, SIOD patients with a deficiency in SMARCAL1 had increased hypermethylation of the *IL7R* promoter, but reduced expression in T cells [26].This is consistent with the results obtained by Marie (Fig. 4).

Globally, approximately 70 mutations associated with the *SMARCAL1* gene are currently described. The exact gene mutations can only be detected in half of SIOD patients. Among them, patients have different genetic backgrounds, but European and American patients comprise the majority of cases. According to an analysis of available data, approximately 90% of mutations associated with the*SMARCAL1* gene have been identified in the Occident and are either truncating or non-truncating mutations. This suggests that the incidence of SIOD may be connected to environmental and genetic factors. Due to limited domestic research on SIOD, and where sufficient knowledge is lacking, this condition can be easily misdiagnosed. In order to lay a foundation for future clinical SIOD diagnosis, further studies on larger populations are required.

In summary, the case of a Chinese patient with mild SIOD associated with a well-known missense mutation and a novel *SMARCAL1*missense mutation is presented. The patient was characterized by a short stature, proteinuria and immune deficiency. This report once more underlines the significance of molecular detection and identification of disease-associated genetic agents. Our findings provide some targeted guidance for the prognosis of this patient. These findings also contribute towards the information available in gene mutation databases.

## Abbreviations

NGS: Next generation sequencing
SIOD: Schimke immune-osseous dysplasia
